# Utilizing metformin to prevent metabolic syndrome due to androgen deprivation therapy (ADT): a randomized phase II study of metformin in non-diabetic men initiating ADT for advanced prostate cancer

**DOI:** 10.18632/oncotarget.28458

**Published:** 2023-06-19

**Authors:** Devalingam Mahalingam, Salih Hanni, Anthony V. Serritella, Christos Fountzilas, Joel Michalek, Brian Hernandez, John Sarantopoulos, Paromitta Datta, Ofelia Romero, Sureshkumar Mulampurath Achutan Pillai, John Kuhn, Michael Pollak, Ian M. Thompson

**Affiliations:** ^1^Division of Hematology and Oncology, University of Texas Health Science Center, San Antonio, TX 77030, USA; ^2^Robert H Lurie Comprehensive Cancer Center of Northwestern University, Chicago, IL 60611, USA; ^3^Roswell Park Cancer Institute, Buffalo, NY 14263, USA; ^4^Institute for Drug Development, Mays Cancer Center at University of Texas Health, San Antonio, TX 78229, USA; ^5^Audie Murphy VA Hospital, San Antonio, TX 78229, USA; ^6^Division of Experimental Medicine, Lady Davis Institute of Medical Research, Jewish General Hospital, McGill University, Montreal, Canada; ^7^Christus Health, San Antonio, TX 78229, USA

**Keywords:** prostate cancer, metformin, metastatic, androgen deprivation therapy, clinical trial

## Abstract

Background: Androgen deprivation therapy (ADT) can lead to metabolic syndrome (MS) and is implicated in ADT-resistance. Metformin showed antineoplastic activity through mTOR inhibition secondary AMPK-activation.

Materials and Methods: To investigate whether metformin mitigated ADT-related MS, we conducted a randomized double-blind phase II trial of metformin 500 mg TID or placebo in non-diabetic patients with biochemically-relapsed or advanced PC due for ADT. Fasting serum glucose, insulin, PSA, metformin, weight and waist circumference (WC) were measured at baseline, week 12 and 28. The primary endpoint was a group of MS metrics. Secondary endpoints include PSA response, safety, serum metformin concentrations and analysis of downstream an mTOR target, phospho-S6-kinase.

Results: 36 men were randomized to either metformin or placebo. Mean age was 68.4. Mean weight, WC and insulin levels increased in both arms. At week 12 and 28, no statistical differences in weight, WC or insulin were observed in either arm. No significant difference in percentage of patients with PSA <0.2 at week 28 between metformin (45.5%) vs. placebo (46.7%). Analysis in the metformin-arm showed variable down-regulation of phospho-S6 kinase.

Conclusions: In our small study, metformin added to ADT did not show a reduced risk of ADT-related MS or differences in PSA response.

## INTRODUCTION

Prostate cancer (PC) is the most commonly diagnosed non-cutaneous malignancy and the second leading cause of cancer related death in U.S. men [1]. Androgen deprivation therapy (ADT) is the backbone of the management of recurrent or metastatic PC. ADT can either be achieved surgically through bilateral orchiectomy or medically through suppression of the Gonadotropin Releasing Hormone (GnRH) axis. Therapeutic efficacy of ADT has clear benefit in PC patients with evidence of improved overall survival (OS) and progression free survival (PFS) when compared to placebo [2–4]. However, one of the unintended consequences of prolonged ADT is the development of metabolic syndrome, leading to an increased risk of cardiovascular disease, diabetes and stroke [5–7]. The most agreed upon criteria for metabolic syndrome are defined by the National Cholesterol Education Program (NCEP), Adult Treatment Panel III (ATP III) [8, 9]. The clinical diagnosis of metabolic syndrome by ATP III criteria requires three of five criteria: elevated waist circumference, elevated triglyceride levels, reduced HDL-C levels, elevated blood pressure and elevated fasting glucose levels [8, 9].

ADT has been shown to be associated with increased fasting glucose levels, elevated serum insulin and insulin resistance, including the diagnosis of diabetes mellitus [10]. It has been shown in rats that the underlying cause of insulin resistance and hyperglycemia is the inability to initiate glycogen synthesis in muscle [11]. This resistance can be reversed by testosterone replacement [12], providing further evidence of the link between ADT and metabolic syndrome. The adverse metabolic profile of PC patients treated with ADT (which suppresses testosterone) has been demonstrated to develop independently of age and BMI [13]. Since secondary consequences of metabolic syndrome include heart disease, diabetes, stroke, metabolic syndrome can lead to significant non-disease related morbidity and mortality [14, 15]. Reports have shown that in men with PC, non-PC related deaths now exceed PC related mortality [16, 17]. Hence, management of the complications of PC-directed therapy may significantly improve quality of life and clinical outcomes.

Metformin is an oral anti-hyperglycemic drug used in the management of type II diabetes that improves overall glucose tolerance [18]. Studies have shown that metformin, through phosphorylation of the AMP-activated protein kinase (AMPK), a major cellular regulator, modulates glucose and lipid metabolism. Phosphorylation of AMPK has been demonstrated to increase muscle glucose transport and inactivate Acetyl-CoA carboxylase (ACC), which serves to inhibit the proximal and rate-limiting step of lipogenesis [19]. There is also evidence that metformin reduces cancer incidence and cancer related mortality in multiple types of cancer [20], thought related to AMPK and AMPK inactivation of ACC.

AMPK is involved in energy homeostasis at both the cellular and macroscopic level. Metformin activates AMPK leading to inhibition of the mTOR pathway by phosphorylating TSC2 and Raptor. Phosphorylation of TSC2 and Raptor subsequently decreases phosphorylation of S6 Kinase 1 (S6K1) resulting in decreased protein and lipid synthesis [21]. Such a result is problematic for cancer cells which typically exhibit the Warburg effect, or glycolysis subsequent increase in lactate production without regard to oxygen concentration [22]. This relegates cancer cells to relying heavily on the use of large amounts of intermediaries (i.e., nucleotides, amino acids, lipids) in the synthesis of the cancer cell biological macromolecules [20, 23]. Furthermore, one study found that metformin might also suppress the growth of androgen-receptor negative PC through induction of autophagy [24]. Therefore, metformin’s ability to phosphorylate AMPK has been demonstrated to provide anti-cancer properties [20, 23], with some properties specific to PC.

We hypothesized that control of increased insulin uptake by overcoming resistance may lead to improved control of metabolic syndrome in PC patients through metrics such as weight, waist circumference and serum insulin levels. We also hypothesized that metformin may enhance ADT’s anti-tumor effects either directly or indirectly through improved control of metabolic syndrome. To test these hypotheses, we conducted a phase II randomized, placebo-controlled, prospective study of metformin vs. placebo in patients with advanced, castrate sensitive PC treated with ADT (NCT:01620593).

## RESULTS

### Patients and treatments

Eligible patients who provided written informed consent were recruited at one center, University of Texas Health Science Center, San Antonio, United States between July of 2011 through July of 2015.

During this period, three randomized phase III trials reported the clinical benefit of adding docetaxel to ADT: Chemohormonal Therapy Versus Androgen Ablation Randomized Trial for Extensive Disease in Prostate Cancer (CHAARTED); [25, 26], Systemic therapy in Advancing or Metastatic Cancer: Evaluation of Drug Efficacy (STAMPEDE); [27] and GETUG-15 [28, 29]. These trials collectively included more than 3000 men with metastatic, castration-sensitive PC. This led to a change in treatment paradigm as ADT+docetaxel became the standard of care for men with metastatic, castration sensitive PC who were eligible for chemotherapy, particularly those with a high metastatic burden. The change in practice at the time of incorporating docetaxel chemotherapy with castration therapy in the management of advanced prostate cancer led to poor enrollment into this study. As a result, this led to a study population that significantly fell short of the power size calculations required as detailed in Methods below. The data reported here reflects the patients that did enter the study despite this change in treatment paradigm and the results that were subsequently found.

**Figure 1:**
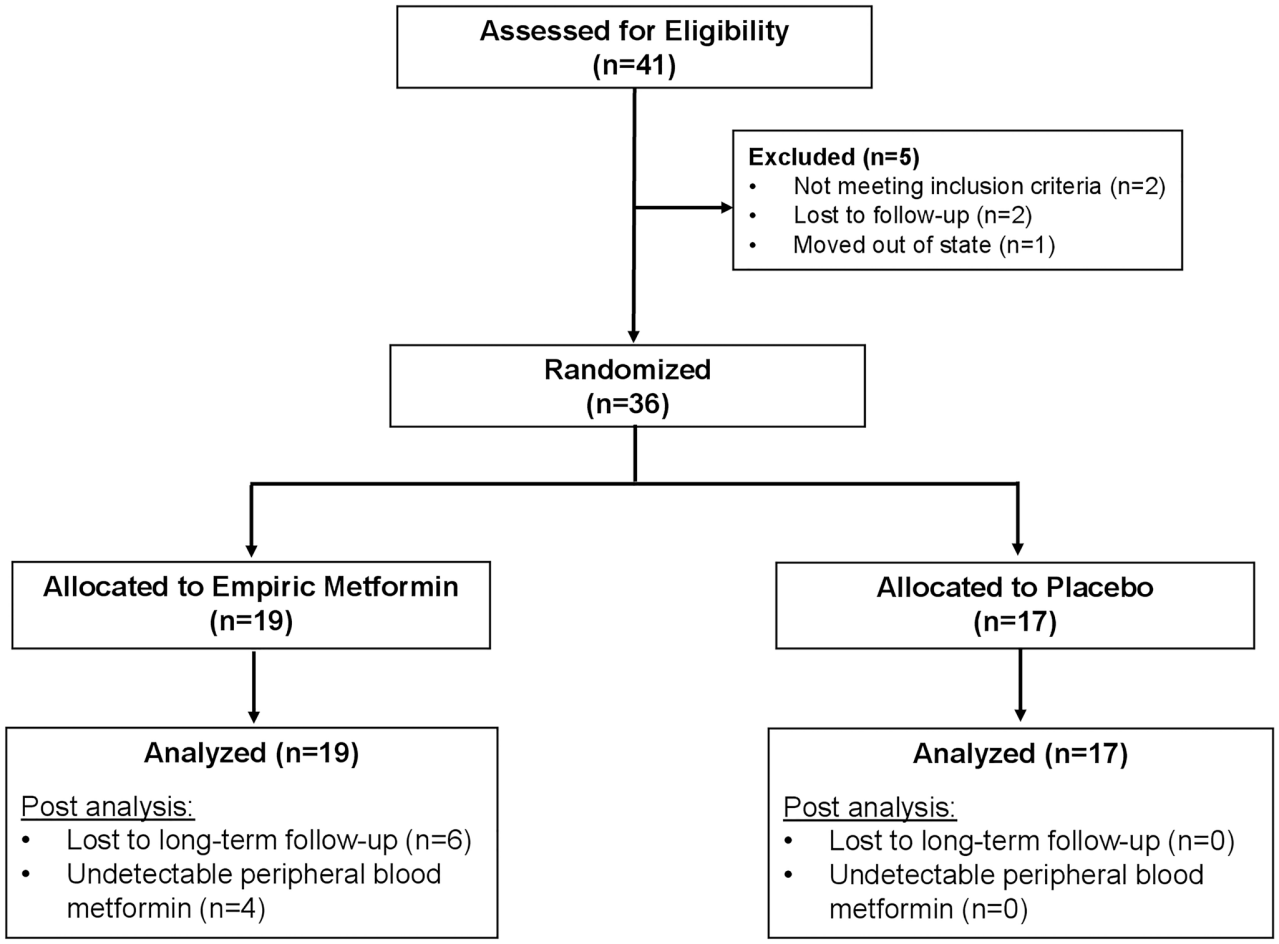
CONSORT diagram: identification, enrollment and randomization of patients.

Forty-one patients signed consent; five patients failed screening. Two of the patients who failed screening were lost to follow up, two patients did not meet inclusion criteria and one patient moved out of the region. Thirty-six patients were randomized from which 19 were allocated to empiric treatment with metformin, the remaining 17 patients were allocated to placebo. All 19 patients in the metformin arm and 17 patients in the placebo arms were included in analysis (Figure 1).

The baseline demographic and disease characteristics, including mean BMI, were well balanced between the two groups (Table 1).

**Table 1:** Baseline patient characteristics

Variable	Level	Metformin group	Placebo group	*P*-value
Total Number		19	17	
Age (years) (mean (SD))		66.90 (10.14)	70.11 (13.54)	0.42^1^
Age (years) (median (range) [61])		66.26 [49.85, 82.27]	72.47 [41.78, 89.31]	0.38^2^
Ethnicity (%)	Non-Hispanic	9 (52.9)	13 (76.5)	0.28^3^
	Hispanic	8 (47.1)	4 (23.5)	
Race (%)	African-American	4 (22.2)	1 (5.9)	0.34^3^
	White	14 (77.8)	16 (94.1)	
ECOG (%)	0	11 (57.9)	9 (56.2)	1^3^
	1	7 (36.8)	7 (43.8)	
	2	1 (5.3)	0 (0.0)	
Stage at Diagnosis (%)	I	2 (13.3)	0 (0.0)	0.11^3^
	II	3 (20.0)	3 (20.0)	
	III	0 (0.0)	4 (26.7)	
	IV	10 (66.7)	8 (53.3)	
Time since original Dx (months) (median, (range))		26.00 (0.00, 165.00)	6.00 (0.00, 125.00)	0.71^2^
PSA at ADT (ng/mL) (mean (SD))		79.41 (129.59)	161.70 (418.08)	0.44^1^
PSA at ADT (ng/mL) (median, (range))		25.00 [0.10, 405.00]	31.95 [0.50, 1680.00]	0.86^2^
Metastatic sites at ADT (%)		7 (36.8)	9 (52.9)	0.19^3^
	Bones	2 (10.5)	4 (23.5)	
	Bones, Liver, Lungs	1 (5.3)	0 (0.0)	
	Bones, Lymph Nodes	5 (26.3)	4 (23.5)	
	Lymph Nodes	4 (21.1)	0 (0.0)	
Progression (%)	Biochemical	2 (40.0)	2 (100.0)	0.43^3^
	Biochemical+imaging	3 (60.0)	0 (0.0)	
BMI (mean (SD))		28.97 (5.06)	26.98 (3.40)	0.19^1^
BMI (median, (range))		29.00 [17.70, 39.30]	26.80 [18.90, 33.10]	0.08^2^

^1^ *T*-test. ^2^Kruskal-Wallis Rank Sum Test. ^3^Fisher’s Exact Test.

As a result of poor accrual, the study was stopped December 2015 and analysis for this study was done prior to the planned interim analysis. Of note, four men randomized to metformin had undetectable serum drug levels despite drug-diary suggesting compliance. These patients were likely pharmacogenomic non-metabolizers of metformin. Table 1 includes results from all patients (including such non-metabolizers) and Supplementary Table 1 removes these four non-metabolizers. Whether or not these patients were included in the study did not significantly impact the metabolic changes seen.

### Metabolic syndrome

**Figure 2:**
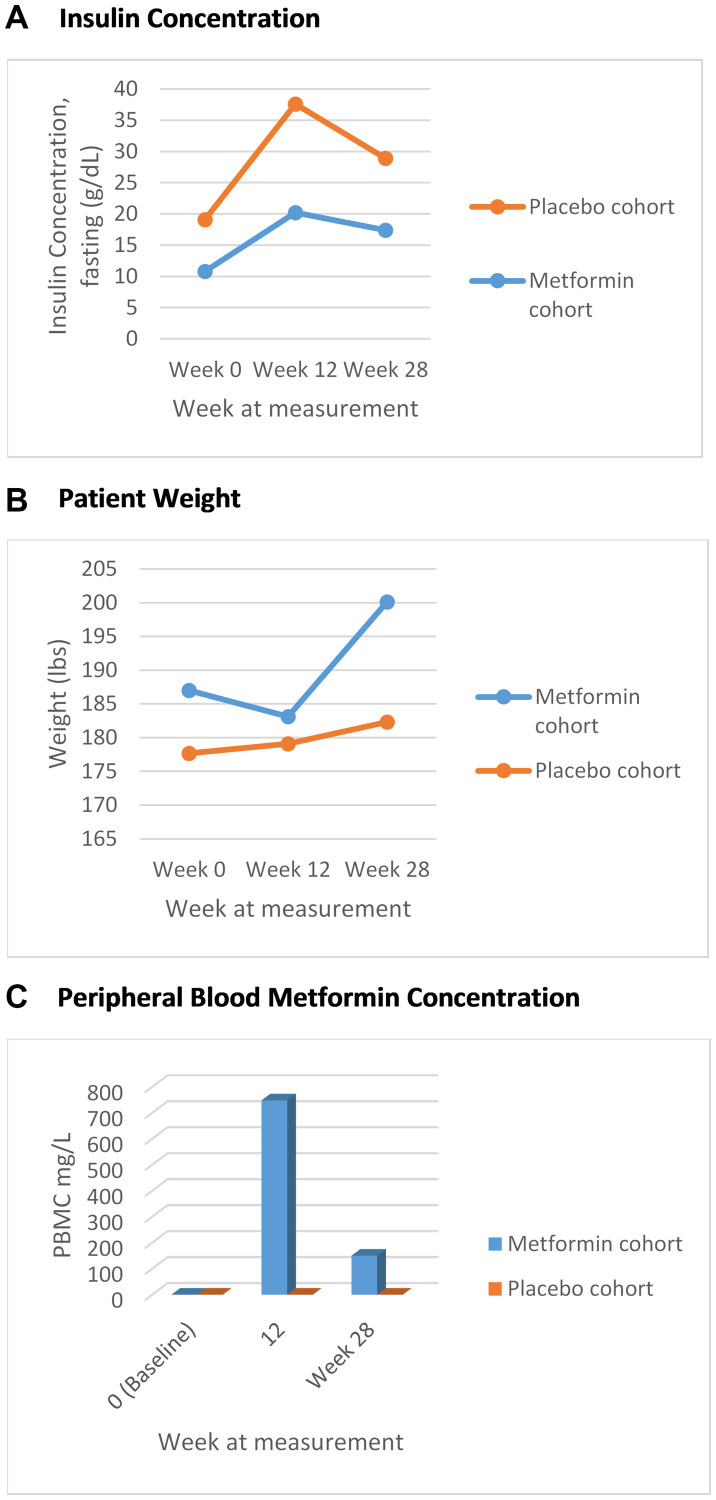
(**A**) Fasting mean insulin concentration over time, marker of metabolic syndrome (g/dL); (**B**) Mean trend in weight, marker of metabolic syndrome (lbs); (**C**) Mean peripheral blood metformin concentration over time.

At baseline, markers of metabolic syndrome including mean weight, WC, serum Insulin concentration in the metformin cohort were 187 lbs, 41.14 cm and 10.03 mIU/L respectively, and 177.65 lbs, 40.52 cm and 8.02 mIU/L in the placebo cohort. An increase in mean weight and serum insulin concentrations were seen across both cohorts (Table 2). At week 28, weight, WC and serum insulin concentration in the metformin cohort increased to 200.1 lbs, 42.1 cm and 17.4 mIU/L respectively (Figure 2A and 2B). In the placebo cohort increases were to 182.3 lbs, 11.5 mIU/L insulin concentration. In the placebo cohort the WC decreased to 37.5 cm (Table 2). At week 12 and 28, there was no statistical difference in markers of metabolic syndrome observed in both cohorts. It is noteworthy that adjustment for change in weight (Delta) across both groups, also was without statistical difference in increase.

**Table 2:** Mean efficacy results in study population

Variable	Metformin group	Placebo group	*P*-value
**Number of patients (*n*)**	19	17	
**Insulin Concentration (g/dL)**			
Week 0 (median (IQR))	8.3 [7.1, 13.5]	7.0 [4.9, 11.5]	0.128^1^
Week 12 (median (IQR))	13.0 [8.0, 19.0]	9.8 [7.7, 19.7]	0.801^1^
Week 28 (median (IQR))	11.0 [7.1, 17.1]	9.2 [6.9, 16.5]	0.733^1^
**Patient Weight (lbs)**			
Week 4 (median (IQR))	189.5 [171.8, 203.5]	183.5 [176.8, 194.0]	0.334^1^
Week 12 (median (IQR))	185.0 [164.0, 198.0]	183.0 [173.8, 192.0]	0.652^1^
Week 28 (median (IQR))	186.0 [182.0, 218.5]	185.0 [173.2, 191.9]	0.429^1^
**Patient Waist Circumference (in)**			
Week 0 (mean (SD))	41.1 (5.3)	40.5 (3.9)	0.696^2^
Week 28 (mean (SD))	42.0 (3.3)	40.6 (4.0)	0.364^2^
**Patient Systolic Blood Pressure (mmHg)**			
Week 4 (mean (SD))	135.7 (20.9)	130.5 (15.9)	0.428^2^
Week 12 (mean (SD))	138.2 (20.8)	136.0 (19.5)	0.757^2^
Week 28 (mean (SD))	133.9 (10.9)	134.2 (18.6)	0.970^2^
**Patient Diastolic Blood Pressure (mmHg)**			
Week 0 (mean (SD))	78.5 (9.5)	77.9 (9.0)	0.853^2^
Week 12 (mean (SD))	77.2 (9.5)	76.1 (8.6)	0.748^2^
Week 28 (median (IQR))	76.5 [70.5, 91.5]	73.0 [70.0, 84.0]	0.350^1^
**Metformin concentration (mg/L)**			
Week 0 (mean (SD))	0.0 (0.0)	0.0 (0.0)	–
Week 12 (mean (SD))	654.5 (858.1)	0.0 (0.0)	–
Week 28 (mean (SD))	104.0 (111.3)	0.0 (0.0)	–

^1^Man-Whitney *U* test. ^2^ *T*-test.

Serum metformin concentration was measured at week 12, week 28, week 40 and week 52 across both placebo and metformin groups. As expected, all patients in the placebo group had a metformin concentration of zero across all measurements (Figure 2C). In the metformin group, at baseline, all patients had a metformin concentration of zero. The trends of increasing weight and decreasing serum fasting insulin concentration was again noted across both cohorts without statistical significance (Table 3), regardless of whether the metformin non-metabolizers were included or not in the analysis (Table 3, Supplementary Table 1). By week 28, 45.5% of the metformin cohort had achieved PSA <0.02 compared to 46.7% in the placebo cohort (*p* = 1.00).

**Table 3:** Delta change in mean measures of metabolic syndrome for all patients^*^

Variable	Metformin group^*^	Placebo group	*P*-value
***n*** (total patients)	19	17	
**Insulin (g/dL)**			
Week 12 (median (IQR))	12.99 [7.96, 19.01]	9.82 [7.68, 19.67]	0.801^1^
Week 28 (median (IQR))	10.95 [7.07, 17.11]	9.22 [6.91, 16.46]	0.733^1^
Delta change (median (IQR))	−4.12 [2.49, 10.44]	−4.35 [2.35, 13.14]	0.807^1^
**Weight**			
Week 12 (median (IQR))	185.00 [164.00, 198.00]	183.00 [173.75, 192.00]	0.652^1^
Week 28 (mean (SD))	186.00 [182.00, 218.50]	185.00 [173.25, 191.95]	0.429^1^
Delta change (median (IQR))	+4.00 [2.25, 9.50]	+3.00 [1.00, 5.00]	0.238^1^
**Number of patients with PSA <0.2 (%) at Week 28**	5 (45.5%)	7 (46.7%)	1.000^2^

^*^Includes 4 patients in the metformin arm who were pharmacogenic non-metabolizers of metformin. ^1^Mann Whitney *U*-test. ^2^Fisher’s Exact test. Abbreviation: IQR: interquartile range.

Eight patients developed adverse events secondary to metformin and had dose reductions (Table 4). All dose reductions were to 500 mg BID dosing, no patients required further dose reductions. At week 12, 4 men randomized to the metformin group had an undetectable serum metformin level. Drug diaries indicated recorded compliance with metformin. Excluding these patients from analysis, markers of metabolic syndrome again did not demonstrate significant change from the mean.

**Table 4:** Adverse events and serious adverse events

Event	Metformin (*n* = 19)	Placebo (*n* = 17)	Total
No. of events	8	2	10
Patients with greater than or equal to 1 adverse events – no. (%)	2 (10.2)	0 (0)	2 (5.5)
Adverse event leading to dose reduction of treatment – no. (%)	7 (87.5)	0 (0)	7 (19.4)
Adverse event leading to withdrawal of treatment – no. (%)	0 (0)	0 (0)	0 (0)
Grade 1 to 3 adverse events – no. (%)	8 (100)	2 (11.7)	10 (100)
Diarrhea	3	1	4
Muscle Pain	1	0	1
Nausea	3	0	3
Increased creatinine	1	1	2

### HOMA-IR

**Figure 3:**
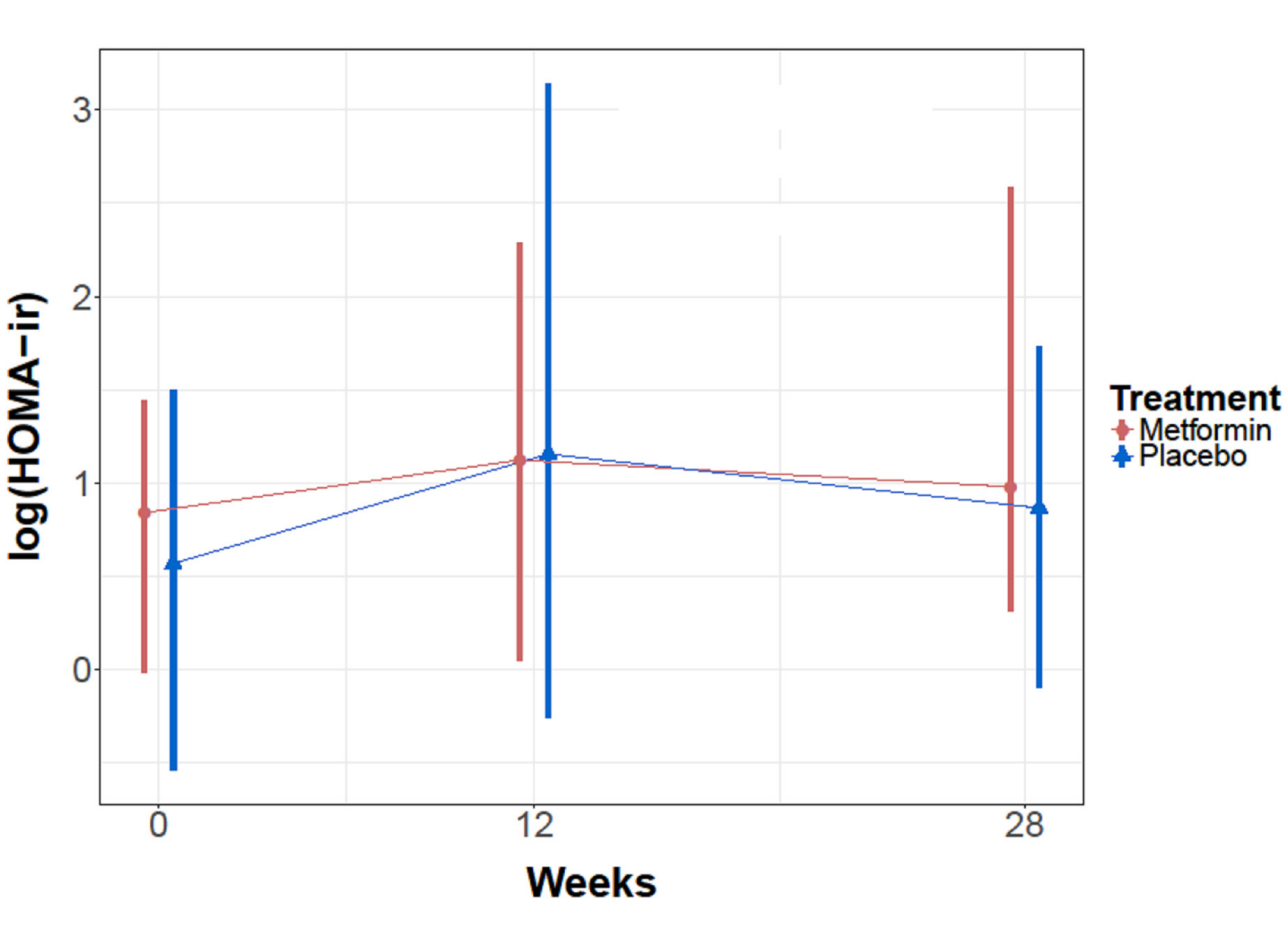
Mean HOMO-ir levels for metformin and placebo cohorts over time.

Homeostatic model assessment is a method for assessing beta-cell function and insulin resistance from basal (fasting) glucose and insulin concentrations [30]. Homeostatic modelling was applied to the data set and the mean HOMO-IR for the metformin cohort at baseline, 12 weeks, 28 weeks was 2.7, 3.8, 3.5 respectively. Whether or not patients with metformin concentration of 0 were excluded from the analysis did not impact the significance of the result. Analysis was performed with a repeated measures linear model with an AR autocorrelation matrix and no significant treatment effects were found (Figure 3).

### PSA efficacy and pharmacodynamic analysis

The median baseline PSA for the placebo and metformin cohort was 31.95 (5.7–77.15) and 25 (6.2–58.7) respectively (*p* = 0.86). Assessing efficacy, 5 patients (45.5%) in the metformin cohort and 7 patients (46.7%) in the placebo cohort achieved PSA <0.2 at week 28. The difference was not statistically significant (*p* = 1.0) and was unaffected by the exclusion of pharmacogenic non-metabolizers of metformin (Supplementary Table 1).

**Figure 4:**
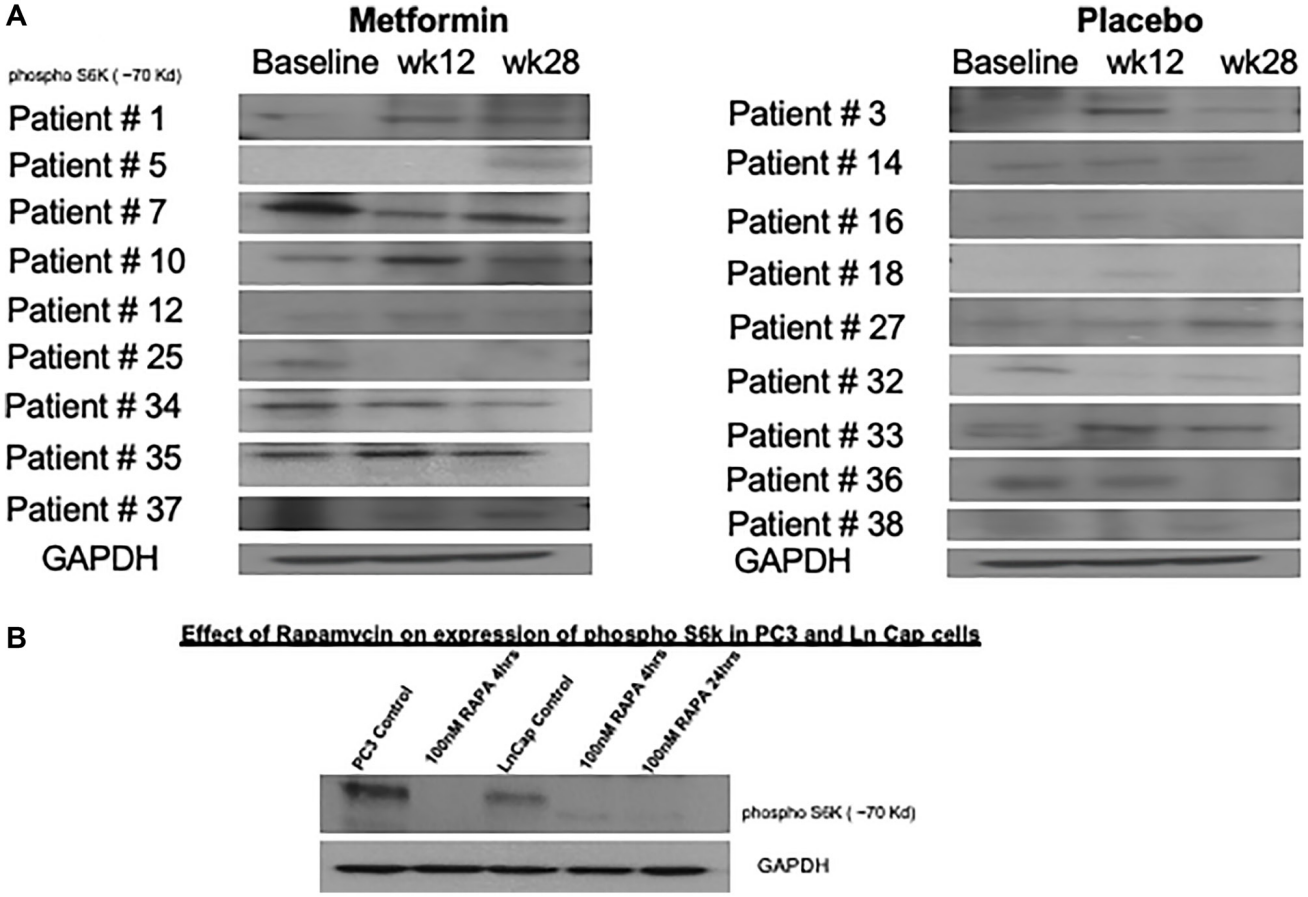
Variable changes in phosphorylation of S6 kinase 1 was observed in both cohorts. (**A**) Peripheral blood mononuclear cells were obtained on all patients enrolled in study at baseline, week 12 and 28. Western blot analysis for phosphorylation of S6 kinase were performed in 18 patients where adequate peripheral blood cell protein was available across all 3 time points. (**B**) As positive controls, PC3 and LnCap prostate cancer cell lines were treated with 100 nM rapamycin and reduction in phosphorylation of S6 kinase shown.

Phospho-S6-kinase levels were analyzed from patient derived blood samples and although there were some downregulation in patients 7, 25, 34 and 37 who received metformin, overall variable regulation of protein levels on western blot in both metformin and placebo cohorts at all time points measured (Figure 4A). The controls using PCa and LnCap PC cells lines did confirm phosphor S6 kinase inhibition following rapamycin treatment (Figure 4B). There was down regulation of phosphor S6 Kinase observed in the metformin treated cohorts. In patients analyzed, the down regulation was evident more in week 40 compared to baseline and 12-week sample.

## DISCUSSION

The results presented are after empiric therapy with metformin compared to placebo to measure its effects on the development of metabolic syndrome in patients with PC on ADT. We caveat that all results in are study are underpowered due to changes in the standard of care as described in Results which limits the generalizability of any conclusions. However, although metformin reduces serum insulin and may have chemoprevention properties, in our small study we found no impact of the addition of metformin to ADT therapy on risk of metabolic syndrome associated with castration therapy and no additional anti-tumor effects.

Multiple studies have demonstrated that markers of metabolic syndrome including weight, WC, BMI and fasting glucose increase shortly after receiving androgen deprivation therapy [14, 31, 32]. Type II diabetes is a consequence of metabolic syndrome and one of the principal defects of type II diabetes is insulin resistance [33, 34]. Long term therapy increases the risk of developing insulin resistance [35, 36]. Metformin is the most widely-prescribed insulin-sensitizing agent in current clinical use and exerts its effect in part by enhancing insulin-stimulated glucose disposal in skeletal muscle [37]. Orchard et al. in a prospective study demonstrated metformin’s ability to decrease the incidence of metabolic syndrome in patients with impaired glucose tolerance but not diagnosed with DM II. Metformin was dosed at 850 mg twice daily and decreased the incidence of metabolic syndrome by 17% compared to placebo [38]. Various studies using metformin tablets of 500 and 850 mg to doses up to 1500 and 2550 respectively have shown that there is a lack of dose proportionality due to the lack of absorption, rather than an alteration in elimination [39, 40]. The standard of care for management of DM II with metformin includes treatment that is begun at a dose of 500 mg twice a day or 850 mg once daily. The dose is gradually increased by 500 mg weekly or 850 mg every two weeks as tolerated and based on the response of the levels of glucose in the blood. The maximum daily dose is 2550 mg given in three divided doses. We therefore standardized treatment in this study and provided 500 mg TID, achieving a daily dose of 1500 mg.

We present a phase II prospective study which randomized patients receiving androgen deprivation therapy to receive metformin compared to placebo and the data (albeit limited by sample size due to explanation in Results) did not show a statistically significant difference in mean markers of metabolic syndrome. We noted a trend towards increase in all categories regardless of randomization to placebo or metformin groups. We expect that serum fasting insulin concentrations would rise in the setting of metabolic syndrome due to insulin resistance and therefore these insulin concentrations were monitored. There was an increase in serum insulin concentrations across both groups but without statistical significance. We investigated delta change in markers of metabolic syndrome and again did not demonstrate statistically significant difference.

As was first described by Matthews et al., we investigated homeostatic modeling in our patient population to quantify insulin resistance and beta-cell function [41–43]. Analysis was performed with a repeated measures linear model with an AR autocorrelation matrix and no significant treatment effects were found between metformin or placebo cohorts. It is possible that we were limited by our sample size. Blood metformin concentrations were measured across all patients. As expected, we did not see metformin positive concentration in any patients in the placebo cohort indicating quality assay. However, two patients at week 12 demonstrated metformin concentration of 0 despite maintaining a drug diary that indicated compliance with treatment. At week 28, those two patients again had metformin concentration of 0, and an additional two patient had metformin of 0 where previously had increased level. While it is possible that these patients had not actually had a treatment of metformin in over 24 hours, the drug diaries that were reported did not correlate with this timing. It is likely that these patients had pharmacogenetic alterations which led them to not metabolize metformin, and their inclusion or exclusion in the study did not affect overall results.

Additionally, serum fasting insulin concentration means remained without significant change upon exclusion of patients with serum metformin concentration of 0. Metformin is excreted renally and does not undergo hepatic metabolism nor biliary excretion. Following oral administration, about 90% of metformin is excreted renally in 24 hours. Lack of proper absorption could lead to decreased concentration of metformin in the setting of reported compliance.

The data in this analysis contrasts with previous studies. A randomized pilot study of ADT and metformin vs. ADT alone, 20 patients in each arm suggested significant improvements in abdominal girth, weight, body mass index and blood pressure was observed, although this study again did not detect changes in biomarkers of insulin resistance between cohorts [44]. It is possible, that the improvement in abdominal girth, weight, body mass index and not the insulin resistant biomarkers, related to the lifestyle changes incorporated with the metformin arm. However, like our current study, the small patient population would suggest larger studies are required before we can draw conclusion the utility of metformin to prevent ADT mediated metabolic syndrome.

Larger studies have demonstrated the potential chemo-preventative effects of metformin on multiple cancers [45, 46]. At present, most studies suggest that hyperglycemia, hyperinsulinemia, IGF-1, DNA damage, inflammatory factors, and obesity may be involved in the pathologic process of diabetes related tumors [47]. In a Finnish study, metformin retrospectively was found to decrease risk for development of PC [48]. The duration of treatment was inversely associated with risk and risk of advanced cancer. Metformin has both chemo-preventative and chemotherapeutic activity which has been studied in pancreatic cancer and demonstrated via down-regulation of specificity protein transcription factors Sp1, Sp3 and Sp4 in pancreatic cancer cells and tumors, accompanied by down-regulation of several pro-oncogenic Sp Regulated genes [49, 50]. The down regulation of pro-oncogenic Sp regulated genes has a role in inhibiting rapamycin (mTOR) signaling and epidermal growth factor (EGFR)-dependent activation of Ras. AMPK activation by metformin has also demonstrated the ability to inhibits acetyl-CoA carboxylase (ACC) and mTOR [20]. AMPK dependent growth inhibition in breast cancer was investigated and the anti-neoplastic effects seen in metformin was demonstrated in breast cancer [51]. Metformin has also demonstrated some efficacy in PC as well [52]. Evans and colleagues demonstrated epidemiological evidence that metformin may be associated with reduced risk of cancer in patients with type 2 diabetes, particularly in patients with extended duration of treatment [46]. Recent clinical benefits of metformin have been studies and retrospective studies have used cancer chemotherapy response and survival time as indicators, proving that metformin has potential clinical benefits [53]. Based on current research, it is hypothesized that metformin combined with radiotherapy and/or chemotherapy can enhance the clinical efficacy against tumors [47].

Recent research has shown that metformin combined with chemotherapy drugs can significantly decrease local recurrence in patients with diabetes and non-small cell lung cancer [54]. In addition, compared with VEGF-A inhibitors alone, metformin combined with VEGF-A inhibitors is more effective in inhibiting tumor growth [55], indicating that the combined application of metformin be a promising route to increase its antitumoral efficacy. To date there have been numerous clinical trials that have evaluated the efficacy of metformin in PC at median doses of less approximately 1 gram (or less) per day with primary objectives of improving recurrence free survival and overall survival [56]. These studies have been conducted in a variety of disease settings (hormone sensitive vs. hormone resistant), including in combination with chemotherapy, radiation therapy, or after surgery, with several ongoing [56].

Despite evidence that metformin activates AMPK leading to inhibition of the mTOR pathway and decreases in phosphorylation of S6 Kinase 1 (pS6K1) [21], in our study, variable reduction of pS6K1 was observed in both metformin and placebo cohorts. Given the low numbers of patients, 9 in each cohort, with adequate blood samples available for analysis in a time dependent manner, no definitive conclusion can be drawn on the utility of blood metformin concentration analysis to assess tumor related mTOR inhibition by metformin. Our results are contrary to other clinical studies that have reported reduction of pS6K1 in patient blood samples in patients with metformin use [57].

PSA was monitored at all intervals of patients in our study of both cohorts, metformin and placebo. Each patient received ADT and patients were started on metformin at the start of ADT. Approximately the same proportion of patients in both the metformin and placebo cohorts achieved PSA <0.02 by week 28. The difference between the two groups was marginal and statistically not significant (*p* = 1.0). In contrast, a previous single arm study of 36 advanced castrate resistant PC men at 10 Swiss centers reported improvement in PSA efficacy and positive effect on metabolic parameters [58]. While to date, there is no large, randomized study that shows evidence of potential anti-cancer role in a non-diabetic population, there is currently an ongoing phase II study evaluating the safety of giving metformin as first line therapy in treatment of patient with locally advanced or metastatic prostate cancer which may provide further insight (NCT01243385).

Adverse events overall were increased in the metformin cohort by comparison to placebo (Table 4). There were increased reports of diarrhea, muscle pain, nausea and vomiting in the metformin cohort. However, in general these adverse events were expected, not serious in nature and nine patients after dose reduction was able to tolerate metformin/placebo on study. There was no reported lactic acidosis in any metformin treated patient in the trial.

## MATERIALS AND METHODS

### Trial Design and oversight

We present a randomized, prospective, double-blind, placebo-controlled phase II trial evaluating the efficacy of empiric glycemic control with metformin in castrated men with advanced PC (NCT:01620593). The trial was designed to enroll a total of 94 men with advanced metastatic PC and men with PC who were candidates for castration therapy despite no evidence of definite metastatic disease including patient with biochemical failure for up to a year. Patients were randomly assigned at a 1:1 ratio to receive either metformin (500 mg TID PO) or placebo.

The randomization phase involved enrollment of patients who had undergone screening for eligibility criteria and had pathologically proven metastatic PC (Figure 1). A computer-generated randomization list was performed by our biostatistician, created with blocks of size 4, and used to assign treatments to patients. Treatments were indicated as ‘A’ or ‘B’. The actual correspondence between these two letters and the active and placebo treatments were not known to clinical investigators or the patient. Patient details including name, ID number and treatment start date were added to this list sequentially by the pharmacy research staff to receive, placebo or metformin paralleled the sequence at which patient were added into the computer-generated randomized list. The pharmacy dispensed the appropriate medication to the patient while ensuring the blinded process was maintained for all patients on study

### Patients

Patients had histologically proven PC and an ECOG performance status of 0 to 2 (on a 5-point scale, with 0 indicating an absence of disability and higher numbers indicating greater disability). Patients required castration therapy with either an LHRH analogue (continuous) or surgical castration. Patient were permitted to use anti-androgen therapy prior to castration; with enrollment into study at the time of castration or within 30 days of castration. All patients were required to have baseline oral glucose tolerance test (OGTT) and patients with values of >200 mg/dL at 2 hours, suggesting a diagnosis of diabetes, were excluded from study and referred for treatment of diabetes. Patients with values between 140 mg/dL and 200 mg/dL indicating impaired glucose tolerance were permitted to enroll and advised dietary modification. Patients with normal values of less than 140 mg/dL were permitted to enroll.

All patients with metastatic disease were documented through computed tomography (CT) or nuclear bone scan, according to Response Evaluation Criteria in Solid Tumors (RECIST), version 1.1. Patients were excluded if they were being treated with any anti-hyperglycemic medications prior to study, had BUN, creatinine, bilirubin levels less than or equal to 1.3 times the upper limit of normal. Safety and dosing adherence were evaluated during each trial visit.

Medical castration using gonadotropin-releasing hormone (GnRH) agonist for all subjects based on treating physician preference was done within 72 hours of an oral glucose tolerance test or baseline visit. Patients were randomly assigned in a 1:1 ratio to receive ADT with metformin (one 500 mg tablet TID) or color matched placebo (the placebo group). Patients were stratified according to the presence or absence of measurable visceral disease, PSA and ECOG performance status score (0 or 1 vs. 2). Metformin or placebo were allowed to be started within 72 hours of medical castration. Men who had previously started castration therapy for metastatic prostate cancer or biochemical flair were also permitted to enter study provided castration therapy was within 4–6 weeks of study entry.

All patients who had not undergone surgical castration, received ongoing ADT to reach or maintain a serum testosterone level of less than 50 ng per deciliter (1.7 nmol per liter).

### Study end points

The primary end point was a group of metrics that reflected the metabolic consequences of ADT including development of hyperinsulinemia and insulin resistance, comparing metformin to placebo in men receiving ADT. Subjects had normal oral glucose tolerance test at baseline. Measurements of metabolic consequences including weight, waist circumference, fasting serum glucose and fasting serum insulin levels were measured in intervals of 4 weeks, results presented at week 12 and week 28. In addition, serum metformin concentration and blood metformin concentration analysis for downstream mTOR protein target inhibition was assessed.

Secondary endpoints included PSA response, defined as a PSA ≤4 ng/ml or PSA <0.02 value at 7 months. A rise of PSA over 25% and PSA ≥2 ng/ml above the nadir required the clinician to repeat PSA again in one month to confirm the further rise and possible treatment failure. Tolerability was followed as a secondary endpoint as is defined by the National Cancer Institute Common Terminology Criteria for AEs (Version 4.0), of metformin and ADT compared to ADT alone.

### Quantification of metformin levels

A liquid chromatography/tandem mass spectrometry (LC-MS-MS) method was implemented for the quantitation of metformin as previously described [59]. Briefly, patient samples were subjected to liquid extraction with acetonitrile. Detection was performed on a Waters triple-quadrupole tandem mass spectrometer in the positive electrospray ionization multiple-reaction monitoring scan mode. The ion transitions monitored were m/z 130.0 → 60.0 for metformin and m/z 260.2 → 116.1 for propranolol (internal standard). The standard curves were linear (*r* = 0.999) over the dynamic range of 5 ng/mL to 3000 ng/mL.

### Pharmacodynamic analysis

Collection of peripheral blood mononuclear cells (PBMCs) were extracted from whole blood in a CPT Vacutainer as recommended by the manufacturer. Briefly, 8 ml of whole blood were collected from patients at baseline, week 12 and 28 and centrifuged at 1500 × g for 20 min at room temperature to isolate the PBMC fraction. The PBMC fraction was then transferred to a 15-ml conical tube, with phosphate buffered saline (PBS) and added PBS to fill the tube and were centrifuged at 600 × g for 10 min at room temperature. After PBS was aspirated, the PBMC pellet was snap frozen and stored at 80°C until use. For analysis, using western immunoblotting was used and probed with phospo-p70S6 kinase and GAPDH antibody (both purchased from Thermofisher).

### Statistical analysis

In a cross-sectional study of men with PC and who received androgen deprivation therapy, the mean fasting insulin was 45.0 mU/mL ± 7.25 mU/mL and a mean HOMA_IR_ of 17.0 ± 2.78 [60]. Assuming a balanced randomized 2-arm study, a mean fasting insulin of 45.0 mU/mL in patients receiving androgen deprivation therapy, a mean fasting insulin of 40.5 mU/mL in patients receiving androgen deprivation therapy and MET (a 10% reduction), a common standard deviation of 7.25 mU/mL, no variation in treatment effect with the presence or absence of metastatic disease, two sided testing of the null hypothesis of equal group means, and a significance level of 5%, this study will achieve a power of 80% with 42 subjects per group with one interim analysis and Obrien Fleming stopping bounds when 50% of patients completed the study. With this design a statistical basis for stopping the study for efficacy will be achieved if the test statistic at the interim analysis exceeds 2.96 (*p* = 0.003051). If the study is not stopped after the interim analysis, the null hypothesis will be rejected for efficacy at the final analysis if the test statistic exceeds 1.96857 (*p* = 0.049002). The final analysis was made once all 94 men were enrolled and data was available for analysis.

Under these assumptions, the same sample size requirement is attained using HOMA_IR_ as the basis for the power calculation. Further assuming 10% lost to follow-up, the required sample size per group is 47 patients (=42/0.9). A 2-sided CI was calculated for mean results in markers of metabolic syndrome including weight, WC, serum insulin concentration. The safety population included all randomly assigned patients who received at least one dose of study treatment. The calculation of homeostatic model assessment for insulin (HOMOir) using the method described by Matthews et al. [41].

### Availability of data and material

The datasets used and/or analyzed during the current study are available from the corresponding author on reasonable request.

## CONCLUSIONS

The present study has numerous strengths including the longitudinal, prospective and placebo-controlled design. The main limitation in assessing the effect of empiric metformin in this study is the limited sample size and variance in criteria to determine metabolic syndrome. Outside of our study, there is evidence to suggest that ADT increases the risk of metabolic syndrome that led to cardiovascular death with prolonged use. For men with PC requiring ADT, many of whom required lifelong ADT, efforts to reduce metabolic syndrome through lifestyle modification including diet and exercise may mitigate some of the risks of ADT. Numerous studies of metformin have been completed in a variety of disease states and settings and future metanalyses may help determine metformin’s true benefit in PC. We anticipate that future larger interventional studies assessing both therapeutic modulation and lifestyle changes will determine whether survival of men with PC requiring ADT can ultimately be improved by this approach.

## SUPPLEMENTARY MATERIALS


