# Correction: Utilizing metformin to prevent metabolic syndrome due to androgen deprivation therapy (ADT): a randomized phase II study of metformin in non-diabetic men initiating ADT for advanced prostate cancer

**DOI:** 10.18632/oncotarget.28530

**Published:** 2023-10-19

**Authors:** Devalingam Mahalingam, Salih Hanni, Anthony V. Serritella, Christos Fountzilas, Joel Michalek, Brian Hernandez, John Sarantopoulos, Paromitta Datta, Ofelia Romero, Sureshkumar Mulampurath Achutan Pillai, John Kuhn, Michael Pollak, Ian M. Thompson

**Affiliations:** ^1^Division of Hematology and Oncology, University of Texas Health Science Center, San Antonio, TX 77030, USA; ^2^Robert H Lurie Comprehensive Cancer Center of Northwestern University, Chicago, IL 60611, USA; ^3^Roswell Park Cancer Institute, Buffalo, NY 14263, USA; ^4^Institute for Drug Development, Mays Cancer Center at University of Texas Health, San Antonio, TX 78229, USA; ^5^Audie Murphy VA Hospital, San Antonio, TX 78229, USA; ^6^Division of Experimental Medicine, Lady Davis Institute of Medical Research, Jewish General Hospital, McGill University, Montreal, Canada; ^7^Christus Health, San Antonio, TX 78229, USA

**This article has been corrected:** In Figure 2A, the insulin concentration graph has been corrected to replace incorrect data due to clerical errors during data entry. The updated Figure 2, produced using the original data, is shown below. In addition, to maintain consistency among mean/median values, the titles of Tables 2 and 3 have been revised, along with the text in the RESULTS section - Metabolic Syndrome paragraph:

## Metabolic syndrome

At baseline, markers of metabolic syndrome including median weight, WC, serum Insulin concentration in the metformin cohort were 187 lbs, 41.1 in and 8.3 mIU/L respectively, and 179.6 lbs, 40.5 in and 7.0 mIU/L in the placebo cohort. An increase in mean weight and serum insulin concentrations were seen across both cohorts from baseline to week 28 (Figure 2A and 2B). At week 28, median weight , WC and serum insulin concentration in the metformin cohort were 186.0 lbs and 11.0 mIU/L respectively (Table 2), and mean insulin and weight were highest at weeks 12 and 28; 14.7 mIU/L and 198 lbs (Figure 2A and 2B). In the placebo cohort, at week 28 median weight and serum insulin were 185 lbs and 9.2 mIU/L respectively (Table 2). At week 12 and 28, there was no statistical difference in markers of metabolic syndrome observed in both cohorts (Table 2). It is noteworthy that adjustment for change in weight (Delta) across both groups, also was without statistical difference in increase (Table 3). The authors declare that these corrections do not change the results or conclusions of this paper.

Original article: Oncotarget. 2023; 14:622–636. 622-636. https://doi.org/10.18632/oncotarget.28458

**Figure 2:**
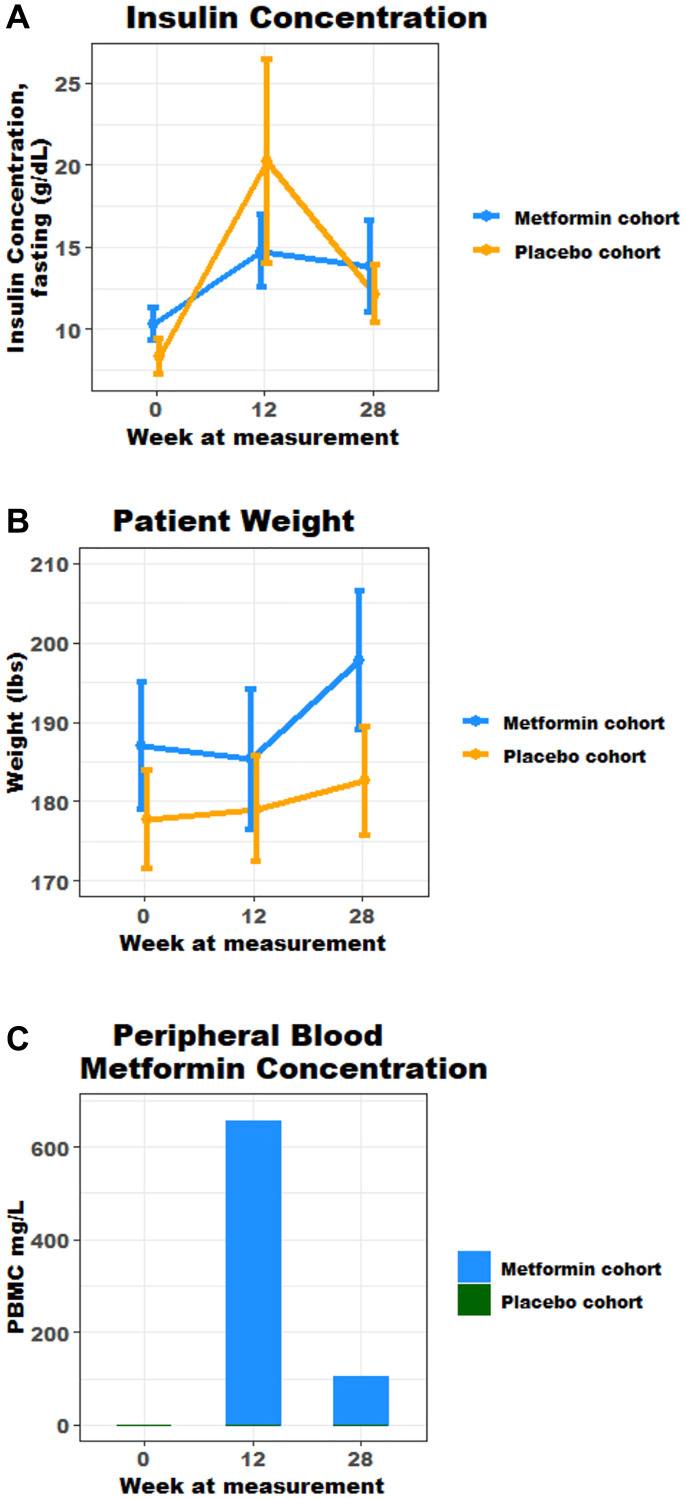
(**A**) Fasting mean insulin concentration over time, marker of metabolic syndrome (g/dL); (**B**) Mean trend in weight, marker of metabolic syndrome (lbs); (**C**) Mean peripheral blood metformin concentration over time.

**Table 2:** Efficacy results in study population

Variable	Metformin group	Placebo group	Overall	*P*-value
***n***	19	17	36	
**Insulin concentration (g/dL)**				
Week 0 (median (IQR))	8.3 (7.1, 13.5)	7.0 (4.9, 11.5)	8.0 (5.9, 12.9)	0.128^1^
Week 12 (median (IQR))	13.0 (8.0, 19.0)	9.8 (7.7, 19.7)	10.8 (7.8, 19.0)	0.801^1^
Week 28 (median (IQR))	11.0 (7.1, 17.1)	9.2 (6.9, 16.5)	10.4 (6.9, 17.2)	0.733^1^
**Patient weight (lbs)**
Week 0 (median (IQR))	187.0 (171.5, 203.0)	179.6 (169.8, 191.5)	185.0 (169.6, 194.0)	0.371^1^
Week 4 (median (IQR))	189.5 (171.8, 203.5)	183.5 (176.8, 194.0)	186.5 (176.2, 197.0)	0.334^1^
Week 12 (median (IQR))	185.0 (164.0, 198.0)	183.0 (173.8, 192.0)	184.0 (173.0, 194.0)	0.652^1^
Week 28 (median (IQR))	186.0 (182.0, 218.5)	185.0 (173.2, 191.9)	185.0 (174.8, 197.5)	0.429^1^
**Patient waist circumference (in)**				
Week 0 (mean (SD))	41.1 (5.3)	40.5 (3.9)	40.9 (4.7)	0.696^2^
Week 28 (mean (SD))	42.0 (3.3)	40.6 (4.0)	41.2 (3.7)	0.364^2^
**Patient systolic blood pressure (mmHg)**				
Week 0 (mean (SD))	136.4 (16.2)	128.2 (16.6)	132.7 (16.7)	0.151^2^
Week 4 (mean (SD))	135.7 (20.9)	130.5 (15.9)	133.2 (18.6)	0.428^2^
Week 12 (mean (SD))	138.2 (20.8)	136.0 (19.5)	137.2 (19.9)	0.757^2^
Week 28 (mean (SD))	133.9 (10.9)	134.2 (18.6)	134.0 (15.4)	0.970^2^
**Patient diastolic blood pressure (mmHg)**				
Week (mean (SD))	78.5 (9.5)	77.9 (9.0)	78.3 (9.1)	0.853^2^
Week 12 (mean (SD))	77.2 (9.5)	76.1 (8.6)	76.7 (8.9)	0.748^2^
Week 28 (median (IQR))	76.5 (70.5, 91.5)	73.0 (70.0, 84.0)	76.0 (70.0, 88.0)	0.350^1^
**Metformin concentration (mg/L)**				
Week 0 (mean (SD))	0.0 (0.0)	0.0 (0.0)	0.0 (0.0)	–
Week 12 (mean (SD))	654.5 (858.1)	0.0 (0.0)	327.3 (683.3)	0.005^2^
Week 28 (mean (SD))	104.0 (111.3)	0.0 (0.0)	56.3 (96.3)	0.005^2^

^1^Man-Whitney *U* test. ^2^ *T*-test.

**Table 3:** Delta change in measures of metabolic syndrome for all patients^*^

Variable	Metformin group^*^	Placebo group	*P*-value
***n*** (total patients)	19	17	
**Insulin (g/dL)**			
Week 12 (median (IQR))	12.99 (7.96, 19.01)	9.82 (7.68, 19.67)	0.801^1^
Week 28 (median (IQR))	10.95 (7.07, 17.11)	9.22 (6.91, 16.46)	0.733^1^
Delta change (median (IQR))	−4.12 (2.49, 10.44)	−4.35 (2.35, 13.14)	0.807^1^
**Weight**			
Week 12 (median (IQR))	185.00 (164.00, 198.00)	183.00 (173.75, 192.00)	0.652^1^
Week 28 (mean (SD))	186.00 (182.00, 218.50)	185.00 (173.25, 191.95)	0.429^1^
Delta change (median (IQR))	+4.00 (2.25, 9.50)	+3.00 (1.00, 5.00)	0.238^1^
**Number of patients with PSA <0.2 (%) at Week 28**	5 (45.5%)	7 (46.7%)	1.000^2^

^*^Includes 4 patients in the metformin arm who were pharmacogenic non-metabolizers of metformin. ^1^Mann Whitney *U*-test. ^2^Fisher’s Exact test. Abbreviation: IQR: interquartile range.

