# Supplementary material for: Utilizing metformin to prevent metabolic syndrome due to androgen deprivation therapy (ADT): a randomized phase II study of metformin in non-diabetic men initiating ADT for advanced prostate cancer
Source: Oncotarget. 2023 Jun 19;14:622–36. doi: 10.18632/oncotarget.28458 (PMC10278660; doi:10.18632/oncotarget.28458)
Supplement: Supplementary file 1 [file oncotarget-14-28458-s001.pdf]

Utilizing metformin to prevent metabolic syndrome due to androgen deprivation therapy (ADT): a randomized phase II study of metformin in non-diabetic men initiating ADT for advanced prostate cancer

SUPPLEMENTARY MATERIALS

Supplementary Table 1: Delta change in measures of metabolic syndrome for metformin metabolizers only**

Variable	Metformin group**	Placebo group	*P*-value
n	15	17	
**Insulin (g/dL)**			
Week 12 (median [IQR])	15.46 [7.96, 22.01]	9.82 [7.68, 19.67]	0.726[1]
Week 28 (median [IQR])	10.36 [7.29, 17.05]	9.22 [6.91, 16.46]	0.655[1]
Delta change (median [IQR])	−4.96 [2.88, 15.80]	−4.35 [2.35, 13.14]	0.929[1]
**Weight (lbs)**			
Week 12 (median [IQR])	177.00 [144.20, 194.08]	183.00 [173.75, 192.00]	0.792[1]
Week 28 (median [IQR])	182.00 [178.50, 224.50]	185.00 [173.25, 191.95]	0.550[1]
Delta change (median [IQR])	3.00 [2.00, 6.00]	+3.00 [1.00, 5.00]	0.598[1]
**Number of patients with PSA < 0.2 (%) at Week 28**	4 (50.0%)	7 (46.7%)	1.000[2]

**Excludes 4 patients who were pharmacogoenic non-metabolizers of metformin with serum levels equal to zero after administration. [1]Mann Whitney *U*-test. [2]Fisher's Exact test.
